# Nursery Assistants' Performance and Knowledge on Cardiopulmonary Resuscitation: Impact of Simulation-Based Training

**DOI:** 10.3389/fped.2020.00356

**Published:** 2020-06-30

**Authors:** Fabien Beaufils, Aiham Ghazali, Bettyna Boudier, Valérie Gustin-Moinier, Denis Oriot

**Affiliations:** ^1^Univ-Bordeaux, Centre de Recherche Cardio-thoracique de Bordeaux, Département de Pharmacologie, CIC 1401, Bordeaux, France; ^2^INSERM, Centre de Recherche Cardio-thoracique de Bordeaux, U1045, CIC 1401, Bordeaux, France; ^3^CHU de Bordeaux, Service d'Exploration Fonctionnelle Respiratoire, Service de Pharmacologie, CIC 1401, Pessac, France; ^4^ABS Lab, Simulation Center, Faculty of Medicine, University of Poitiers, Poitiers, France; ^5^Emergency Department and Emergency Medical Service, University Hospital of Bichat, Paris, France; ^6^Early Childhood Department of the Cityhall Social Action Center of Poitiers, Poitiers, France; ^7^Pediatric Emergency Department, University Hospital of Poitiers, Poitiers, France

**Keywords:** infant, CPR, simulation-based training, nursery assistants, assessment, performance

## Abstract

**Background:** Child cardiac arrest is rare, but more frequent among infants, requiring immediate cardiopulmonary resuscitation (CPR). Many studies have reported that simulation-based training (SBT) increased CPR performance of healthcare providers. However, the CPR performance of laypeople using basic life support remains poorly known. The aim of this study was to assess nursery assistants' (non-healthcare providers) CPR performance and knowledge, before and after SBT.

**Methods:** The study was carried out from January to June 2018 in the city of Poitiers, France. Two teaching sessions (T1 and T2) and two evaluation sessions (E1 and E2) were performed. Performance in infant CPR on a manikin at E1 and E2 were videotaped and assessed automatically with Resusci Baby QCPR® and a SimPad PLUS SkillReporter (QCPR Global Score and skills) and by an observer using an original CPR performance checklist (MCPR Global-Score and skills). Nursery assistant's CPR knowledge was assessed by a questionnaire at the beginning and the end of the session T1, E1, and E2.

**Results:** Twenty-Seven nursery assistants over 30 contacted were included. There was an improvement between E1 and E2 in QCPR Global-Score (E1: 42.4 ± 23.6 vs. E2: 55.1 ± 23.7%, *p* = 0.032), MCPR Global-Score (E1: 50.0+11.9 vs. E2: 72.3+8.5%; *p* < 0.001) and theoretical knowledge with score (over 45) of 16.9+5.4 before T1 and 35.2+2.7 after E2, respectively (*p* < 0.001). The improvement mainly concerned QCPR and MCPR compression steps scores. MCPR Global-Score was strongly correlated to QCPR Global-Score (*r* = 0.61; *p* < 0.01) and predictive to CPR quality determined by QCPR Global-Score (AUC = 0.77; *p* < 0.01) with a high sensitivity and negative predictive values. Moreover, these improvements were maintained 2 months after training with no difference between scores obtained by the three groups 15, 30, or 60 days after simulation-based training session T2.

**Conclusion:** SBT could significantly improve knowledge and skills in infant CPR management by nursery assistants especially for chest compression. CPR performance checklist appeared as an interesting tool to assess CPR performance quality.

## Introduction

Pediatric prehospital cardiac arrest are rare, with an overall incidence of 8 to 10 per 100,000 persons (1–3) but occurs mostly non-public location such as the residence (96% for infants) (4). The survival is low by about 10% (5). Survival can be improved by quality cardiopulmonary resuscitation (CPR) performed immediately (3, 6, 7). However, CPR performed by laypeople is regularly suboptimal (8, 9), initiated in only half of OHCA (10) and more frequently when bystanders were previously trained in CPR (10).

To improve quality CPR of rescuers, the use of simulation-based training (SBT) increased these last years and has proven its efficiency to improve CPR skills and maintain them over time (11–13). Moreover, development of specific devices (QCPR devices) on the top of manikins (14–16) coupled with SBT allowed to easily and objectively assess CPR quality, giving details on CPR performance. However, these devices and most of the interventions trying to improve CPR performance with SBT focused on healthcare providers but not on laypeople (17, 18) while they could represent the first possible rescuers in OHCA.

Because infant OHCA are more frequent than in children (1), occur more frequently at home and have a lower survival rate than in adults (19), in France, an initial CPR training including infant CPR is mandatory for nursery assistants certification (20) and then regular CPR training is recommended. However, effectiveness of CPR training to improve infant CPR performed by nursery assistant has not been regularly assessed. While previous study had demonstrated that after traditional basic life support courses, skills retention gradually decreased (21) and that could contribute to the low rate of survival after OHCA (22).

We hypothesized that SBT with mastery training would increase infant CPR performance of nursery assistants.

The aim of this study was to assess infant CPR performance among a population of nursery assistants working alone at home, before and after mastery training including didactics and SBT. The primary objective was to improve nursery assistants' infant CPR performance quality. Secondary objectives were: (1) To assess CPR specific steps quality; (2) To measure theoretical test scores at different times of the study; (3) To compare theoretical scores to CPR performance scores; (4) To assess maintaining of skills and knowledge over time.

## Methods

### Population

This monocentric prospective pilot study with assessment before and after intervention took place in Poitiers, France, from January to June 2018 and involved nursery assistant (NA), managed by the “Early Childhood department” of the City hall of Poitiers, working alone at home. After the agreement of the “Early childhood department,” the 30 nursery assistants were contacted for the study. Inclusion criteria were: being a certified NA (involving previous participation in certified CPR training), having participated in CPR training within the last 2 years and giving a written informed consent to participate. Participants who did not completed all the study procedure were excluded. The study received the approval of the simulation center and the local ethics committee of the Faculty of Medicine of Poitiers.

### Protocol

**Figure 1:**
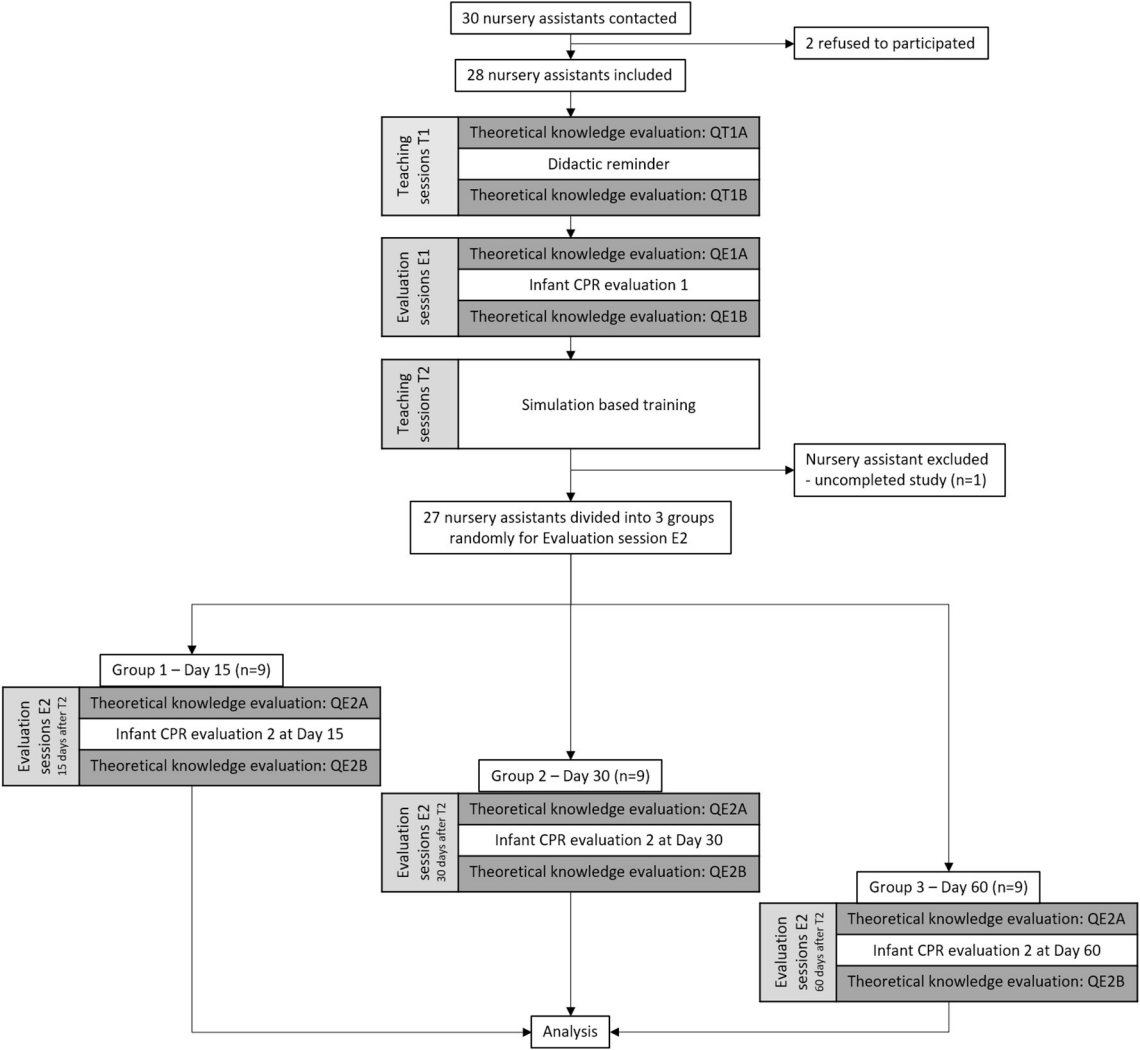
Study design and flow chart.

The study design is represented in Figure 1. Briefly, nursery assistants participated to two teaching sessions (T1 and T2) and two evaluation session (E1 and E2). T1 consisted in a 1-h didactic reminder about infant CPR (T1) performed by CPR-certified physician instructors (FB, AG, DO) explaining the epidemiology of cardiac arrest in infant and detailing the steps of CPR according to the European Resuscitation Council recommendation (23). T2 consisted in SBT focus on infant CPR training using mannikin (BabyBen®, Laerdal, Norway) under supervision of CPR-certified physician instructors (FB, AG, DO). The duration of T2 was 2 h allowing participants to rehearse and correct their performance. Each participant repeated the CPR sequence on the manikin a minimum of three times. For evaluation sessions, the participant had to perform an infant CPR on manekin. The performance was assessed automatically and with a checklist as described below. E1 was performed before SBT and E2 after SBT training performed at T2. For E2, participants were randomly divided into three equal groups and the session was performed 15 (Group 1 – D15), 30 (Group 2 – D30), or 60 days after T2 (Group 3 – D60).

Except for T2, knowledge test was performed at the beginning and the end of each session. Content of the scenario was explained out to participants during briefing just before simulation and of the simulation room. The same scenario (Appendix A1) was used for evaluation session to allow comparison between scores. To improve the realism of the situation, the evaluation room has been converted into a baby's room and contained the manikin (dressed like a baby and lying in a crib), towels, table and phone (to simulated the call for help to the emergency medical system. For each evaluation, the performance starts immediately after entering the room where the manikin is located and duration was limited at 4 min. All performances were videotaped and followed by a good-judgment debriefing (19) run by a trained facilitator (FB, DO) including a short feedback on participant's performance to improve further CPR practice. The study was performed in addition of the usual CPR training. However, to limit bias, all of the study was performed between two session of the usual CPR training.

### Performance Measure

Basic life support (BLS) competencies were measured using Resusci Baby QCPR® (ref: 161-01250, Laerdal Medical) and a SimPad PLUS SkillReporter (ref: 206-30033, Laerdal Medical) connected defining a QCPR Score, a QCPR Breathing Score (based on the time of the first recue breath, tidal volume, and respiratory rate) and a QCPR Compression Score (based on time of the first compression, compression rate, compression depth, chest recoil, finger position, and no flow duration). Scores were expressed by the device as percentage of success. According to manufacturer instructions, the CPR performance quality was determined by the QCPR scores which reflect a basic (<50%), intermediate (≥50 and <75%), or advanced (≥75%) CPR performance quality. A CPR performance checklist created for the study based on the European Resuscitation Council recommendations (Appendix A2), was also used to measure basic life support competences not recorded with the skill reporter. The checklist was composed by 18 items (e.g., check breath, start rescue breaths, start chest compression) and were associated to a 2-point (0 or 1 point) or 3-point (0, 1 or 2 points) scale. The sum of all items scores defined the MCPR Global-Score. Two independent evaluations, spaced by 6 months, were performed by the referring observer (FB) for each CPR performance (one during evaluation sessions and the other, blinded of the first results, using anonymized participants' performance videotapes) and intra-class correlation was calculated.

The same QCPR Global-Scores thresholds were used for MCPR scores to determine CPR quality.

#### Theoretical Knowledge Evaluation

A written theoretical knowledge test based on questionnaire created for the study was used to measure infant CPR knowledge and included 36 items (Appendix A3) for a maximum score of 45. The questionnaire was composed by 7 sections: Infant ALTE signs (/7 points), Cardiac arrest generalities (/6 points), Infant cardiac arrest management (/6 points), Alone rescuer CPR (/6 points), Ventilation management score (/4 points), chest compression management (/5 points) and Emergency call section (/11 points). Each correct answer per item corresponds to 1 point except for 2 items scored out of 2 points and 1 out of 3 points (3 answers expected). For the Alone rescuer CPR section, corresponding to one item, participant had to order CPR step for a maximum of 6 points. Throughout the study, each participant had to complete 6 questionnaires (QT1A, QT1B, QE1A, QE1B, Q2EA, QE2B) (Figure 1). Before analysis participant's answers were double check by investigator (FB, DO).

### Statistical Analysis

Statistical analysis was performed with SPSS 26.0 (SPSS Inc., Chicago, IL). Results are presented as mean + standard deviation (SD) or median with interquartile range [IQR_25_-IQR_75_]. Comparisons of continuous variables were made with paired *t*-test or ANOVA for paired and parametric values, Wilcoxon or Friedman tests for non-parametric paired values and Mann–Whitney or ANOVA test for non-parametric and unpaired values. Categorial variables were analyzed with Chi square test or Fisher's test. Pearson test was used to assessed correlation for parametric values or Spearman test for non-parametric values. Bland and Altman test was used to assess intra-observers reproducibility. A *p*-value < 0.05 was considered significant.

## Results

### Population

Out of the thirty contacted nursery assistants, two refused to participate, and one was excluded because she did not complete the study protocol (Figure 1). Characteristics of the population are presented in Table 1. The three groups obtained for E2 did not present significant difference.

**Table 1:** Characteristics of the population.

	**Total**	**Group 1 D15**	**Group 2 D30**	**Group 3 D60**	***p*-value**
Number of participants	27	9	9	9	
Age at onset of the study (years)	51.0 [49.0;56.0]	51.0 [49.5;59.0]	52.0 [48.5;54.5]	49.0 [47.0;54.0]	0.214
Past hospital experience (yes/no)	3/24	1/8	1/8	1/8	1.000
Time since beginning of the practice (years)	18.0 [14.0;25.0]	25.0 [13.5;29.0]	16.0 [14.0;19.0]	17.5 [15.5;23.8]	0.524
Number of children taken in care	2 [1;4]	2 [1;3]	2 [1;3]	2 [1;3]	0.572
Experience of previous CPR training (yes/no)	27/0	9/0	9/0	9/0	1.000
Time since last CPR training (years)	1.4 [1.0;1.8]	1.3 [1.3;1.5]	1.4 [0.8;1.8]	1.4 [1.0;1.8]	0.658
Number of CPR training since beginning of the practice	5.0 [4;7]	4.5 [1.8;5.8]	4.0 [1;10]	5.5 [5;9.3]	0.413
Stressed to participate in this study (yes/no)	4/23	1/8	2/7	1/8	0.587

*Group 1 D15, Group 2 D30, Group 3 D60 corresponding to nursery assistant participation in session E2 at 15, 30 and 60 days after session T2 respectively. Data are median [IQR_25_; IQR_7_5]. Comparisons of continuous variables were made with ANOVA test or with Kruskal–Wallis tests for non-parametric variables. Categorial variables were analyzed with Chi square test*.

### Practical Evaluation

#### Improvement in CPR Performance: Global-Scores

While the QCPR Global-Score was significantly improved between E1 and E2, the increase in the number of participants with a QCPR Global-Score between 50 and 75% or over 75% was not significant (Table 2).

**Table 2:** CPR performance global scores.

	**Session E1**	**Session E2**	***p*-value**
Number of participants	27	27	
QCPR Global-Score (%)	42.4 ± 23.6	55.1 ± 23.7	0.032
Participants with QCPR Global Score (yes/no)			
[0 to 50% [	16	12	0.414
[50 to 75% [	8	7	1.000
[75 to 100% [	3	8	0.175
MCPR Global-Score %	50.0 ± 11.9	72.3 ± 8.5	<0.001
MCPR Global score /25	12.5 ± 3.0	18.1 ± 2.1	<0.001
Participants with MCPR Global Score			
[0 to 50% [	12	0	<0.001
[50 to 75% [	15	18	0.577
[75 to 100% [	0	9	<0.001

*QCPR Global-Score represented CPR performance score assessed automatically with SimPad PLUS SkillReporter. MCPR Global-Score represented CPR performance score assessed manually CPR performance grid*.

*Data are means ± standard deviation for quantitative values. Comparisons of continuous variables were made with Paired T-test for parametric variables or Wilcoxon test for non-parametric variables. Categorial variables were analyzed with Fisher's exact test*.

Considering MCPR scores, intra-observer agreement and intra-class correlation over time were high and lack of intra-observer agreement over time was low at E1, E2 or for all observations together (Figures E1A,B). MCPR Global-Scores and the number of participants with a score between 50 and 75% or over 75% were increased significantly between E1 and E2 (Table 2). QCPR and MCPR Global-Scores appeared strongly correlated at E1 and E2 or with all values together (Figures E2A–C). In addition, ROC curves analysis performed to determine the ability of MCPR Scores to predict a QCPR Global-Score had significant AUCs and >0.5 with a high sensitivity and a high negative predictive value (NPV) to predict QCPR Global-Score ≥50% and a high specificity and a high negative predictive value (NPV) to predict QCPR Global-Score ≥75% (Table E1).

#### Changes in Specific BLS Step of CPR (Table 3)

##### Airways and communication

There was a significant improvement in participants positioning the manikin in neutral position, opening airways, inspecting airways and performing CPR during the call to emergency medical system. There was no significant change for stimulating the manikin, putting it on hard surface and calling emergency medical system item performed by most of participant at E1.

**Table 3:** BLS steps.

	**Session E1 *N* = 27**	**Session E2 *N* = 27**	***p*-value**
***AIRWAYS step***			
Stimulate infant	24/3	25/2	1.000
Check breath	12/15	17/10	0.275
Put on hard surface	26/1	27/0	1.000
Put in neutral position	2/25	20/7	<0.001
Opening airway	8/19	17/10	0.028
Inspecting airways	0/27	13/14	<0.001
***COMMUNICATION step***			
Calling emergency medical system	23/4	27/0	0.111
Continue chest compression during call	4/23	22/5	<0.001
***BREATHING step***			
QCPR Breathing Score (%)	62.4 ± 27.4	58.4 ± 21.7	0.367
Participants with QCPR Breathing Score			
[0 to 50% [	4/23	2/25	0.387
[50 to 75% [	14/13	18/9	0.268
[75 to 100% [	9/18	7/20	0.551
Time of first rescue breath (sec)	60.7 ± 46.0	51.0 ± 22.9	0.732
Mean tidal volume administered (ml)	69.9 ± 65.7	92.4 ± 67.5	0.148
Ventilation with correct volume (%)	22.7 ± 24.3	12.3 ± 15.11	0.067
Volume over the maximum volume (%)	52.3 ± 37.5	71.1 ± 33.9	0.052
Volume below the minimum volume (%)	25.0 ± 31.5	16.7 ± 28.4	0.148
Starting ventilation before chest compression (yes/no)	22/5	27/0	0.019
5 Rescue breaths before chest compression (yes/no)	3/24	25/2	<0.001
Rescue breaths with long duration (yes/no)	3/24	3/24	1.000
Chest expansion observed during rescue breathes (yes/no)	13/9	24/3	0.022
***COMPRESSION/CIRCULATION step***			
QCPR Compression Score (%)	31.6 ± 35.9	53.3 ± 53.3	<0.001
Participants' QCPR Compression Score (Yes/No)			
[0 to 50%]	18/9	12/15	0.010
[50 to 75%]	5/22	4/23	0.807
[75 to 100%]	4/23	11/16	0.033
Time of first compression (sec)	71.0 ± 34.5	65.9 ± 17.8	0.572
Compressions rate/min	101.1 ± 23.1	105.6 ± 15.7	0.200
Compressions with correct rate (%)	27.9 ± 29.8	48.5 ± 30.3	<0.001
Participant with rate between 100 and 120/min (Yes/No)	7/20	17/10	0.013
Compressions with correct hand position (%)	66.3 ± 32.7	73.4 ± 28.0	0.280
Compressions with correct depth (%)	68.5 ± 37.0	68.1 ± 35.7	0.594
Chest compressions correctly recalled (%)	81.2 ± 24.6	79.2 ± 25.0	0.638
No flow duration during the session (sec)	93.7 ± 41.3	81.3 ± 19.7	0.119
No flow duration during the session (%)	43.4 ± 16.9	37.8 ± 10.3	0.199
No flow duration after the first compression (sec)	22.7 ± 12.1	15.5 ± 6.2	0.006
Starting chest compressions (Yes/No)	26/1	27/0	1.000
Maintaining airway in open position (Yes/No)	2/24	6/21	0.250
Using two fingers technique for compressions (Yes/No)	10/16	20/7	0.013
With good position of finger on chest (Yes/No)	12/14	18/9	0.170
Correct chest dept (Yes/No)	16/10	23/4	0.066
Performing compression with rate			
<80 or > 140/min (Yes/No)	10/17	3/24	0.026
[80–100[or]120–140]/min (Yes/No)	13/14	13/14	1.000
[100–120] /min (Yes/No)	3/24	11/16	0.013
Performing compressions-breath 30:2 (Yes/No)	16/10	26/1	0.002

*QCPR Breathing-Score and QCPR Compression-Score represented scores assessed automatically with SimPad PLUS SkillReporter*.

*Data are means+standard deviation for quantitative values. Comparisons of continuous variables were made with Paired T-test for parametric variables or Wilcoxon test for non-parametric variables. Categorial variables were analyzed with Fisher's exact test*.

##### Breathing

There was no significant difference of QCPR B-Score between E1 and E2, however the number of participants starting and performing 5 rescue breathes before chest compressions and with chest expansion observed during rescue breathes. The time of the first rescue breath after the discovery of the manikin was decreased at E2 compared to E1 but the difference was not significant. The percentage of ventilation administering the correct volume remained very low at E2, with most of the volumes administered exceeding the maximum expected volume.

##### Circulation/compression

QCPR C-Score and number of participants with a QCPR C-score over 75% increased significantly between E1 and E2. Moreover, the percentage of compressions performed at a correct rate, the number of participants with an average compression rate of 100–120 per minute, and the use of the two fingers technique for chest compression and performing 2 rescue breathes each 30 chest compressions increased significantly between E1 and E2. The time to first compression and the percentage of no flow duration were decreased between E1 and E2 but the difference was not significant. However, the no flow duration after the first compression decrease significantly between the session. Chest compression depth and recoil, or correct hand position were not different between E1 and E2.

### Theoretical Knowledge Evaluation

**Figure 2:**
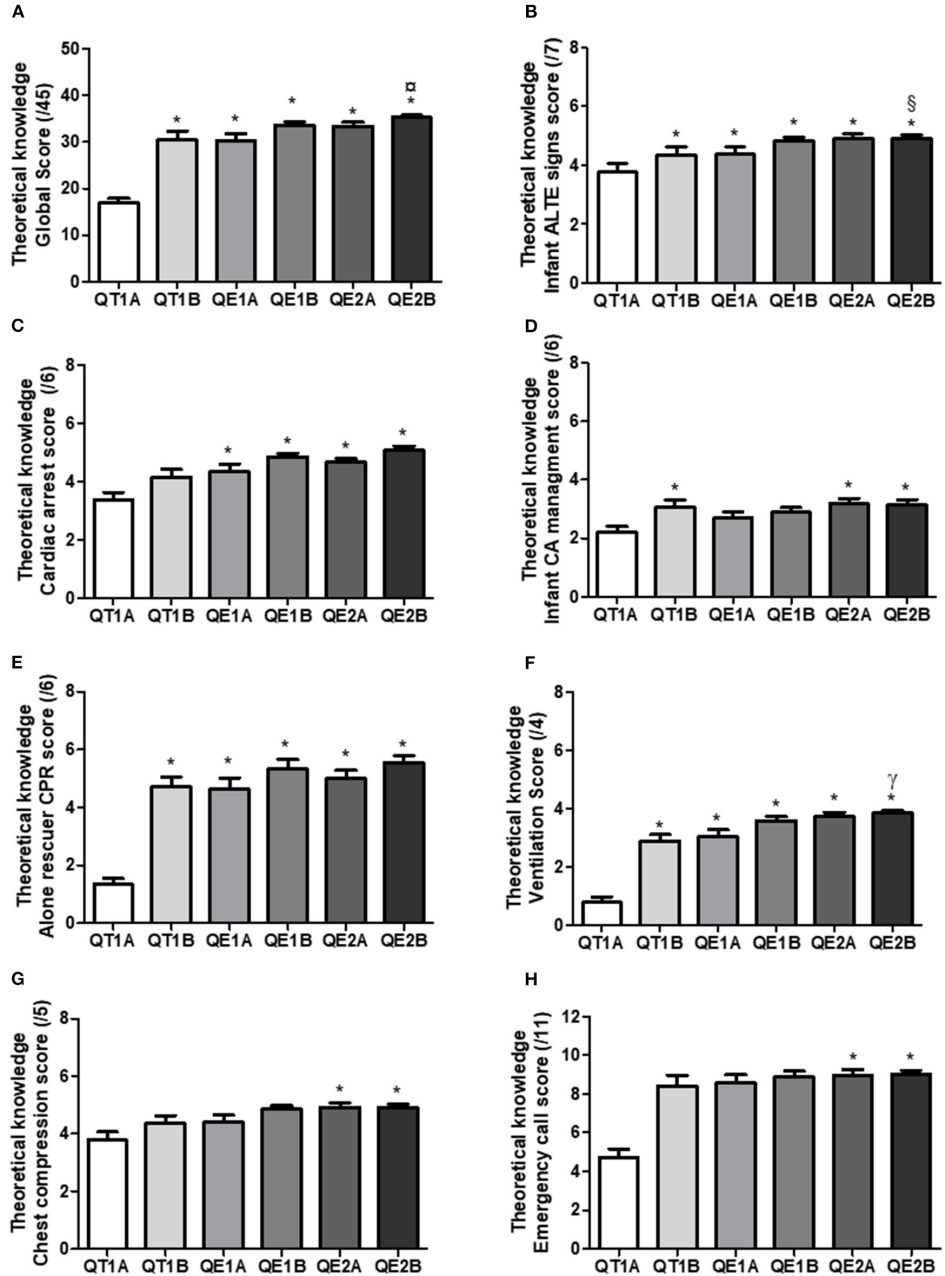
Theoretical knowledge score. Theoretical knowledge was assessed with questionnaire at the beginning and the end of T1, E1, and E2 defining qussssestionnaire T1A, T1B, E1A, E1B, E2A, and E2B respectively to determine the evolution of **(A)** the theoretical knowledge global score composed by **(B)** infant ALTE signs score, **(C)** cardiac arrest generalities score, **(D)** infant cardiac arrest management score, **(E)** alone rescuer CPR score, **(F)** ventilation management score, **(G)** chest compression management score and **(H)** Emergency call score and. *P* < 0.05 * vs. T1B, ^¤^ vs. E1A, ^§^ vs. E2A, ^γ^ vs. T1A.

Score obtained before any intervention at the beginning of T1 (QT1A) increased significantly after didactic intervention (QT1B) with an improvement of knowledge in infant cardiac arrest management, order of CPR steps of management, chest compression, ventilation, and emergency call (Figures 2A–F) and after E1 for infant ALTE signs and cardiac arrest score (Figures 2G,H). Then scores were not significantly different. Interestingly, there was a correlation between theoretical knowledge score at the beginning of E1 (QE1A) with QCPR or MCPR Global-Score at E1 (Figures E3A,B) but not between theoretical knowledge score at the beginning of E2 (QE2A) with QCPR or MCPR Global-Score at E2 (Figures E3C,D).

### Evaluation of Persistence of Performance Over Time

BLS competencies assessed with Skill Reporter or with BLS checklist and QE2A questionnaire score were not significantly different between the three groups performing E2 15, 30, or 60 days after SBT training session T2 (Table E2).

## Discussion

### Main Results

This simulation-based study demonstrated an improvement in infant CPR performance, skills and knowledge—especially concerning Chest compression steps—with a satisfactory retention at 15, 30, or 60 days in a population of nursery assistants. Scores assessed with checklist were strongly correlated to automatically assessment and predictive of the of CPR performance quality determined by skill recorder.

### Discussion of Primary Outcomes

In this study, most of participants had CPR performance scores and Compression scores above 50% at baseline while they were previously trained to CPR, suggesting that first aid management of cardiac arrest training was insufficient and that scores of untrained laypeople would be similar or worse (24). Most simulation studies included healthcare providers and did not address the infant OHCA cardiac arrest. However, some studies have shown that knowledge of first aid management was a predictor of parents' self-efficacy in infant CPR skills (25, 26). The present study showed the benefits of SBT combined with didactics corresponding to mastery training as previously reported (27) and demonstrated the ability of SBT to improve CPR performance even in lay people such as nursery assistants (13, 21, 28–30). This improvement can be observed both with automatically assessment using QCPR device or by observer assessment using CPR checklist. However, a QCPR Global-Score over 75%—corresponding to advanced CPR quality—was not obtained by all the performers. Nevertheless, a study performed with healthcare providers (31) or with laypeople reported similar results (32). We could imagine that a single practical training course would be sufficient to improve a low starting CPR quality to an intermediate one, and only a repeated training would be necessary to achieve an advance CPR quality. Another explanation from this lack of results could be related to the difficulty for some participants to maintain the same CPR quality for 4 min of simulation because of exhaustion since it was a population close to 50 years old.

### Discussion of Secondary Outcomes

As previously demonstrated, mastery training, led to an improvement in participants' skills and knowledge in different steps of infant CPR management (13, 16, 17). In this study, participants improved their skill in each step of infant CPR.

Surprisingly QCPR B-Score was not increased after SBT. This lack of result could be explained by difficulties for participants to administrate a correct volume during rescue breathes. Previous studies highlighted that rescue breathes in CPR did not improve survival or morbidity (33–35) except for infants (up to 1 year) because of hypoxic high frequency of CA. However, to improve survival or morbidity, these studies concluded that ventilation must be optimal—that was not the case even after CPR training. Moreover, the first chest compression was delayed over 1 min representing a considerable no flow duration even if after the first compression the no flow duration was low. This encourages, for laypeople, to perform infant CPR with a C-A-B sequence to initiate sooner chest compressions and reduce no flow duration as mentioned by the American Heart Association guidelines or European Resuscitation Council Guidelines for adults (23, 34, 36). Moreover, as reported, in infants, to not perform rescue breath would not be associated with several outcomes 1 month after CPR (35, 37).

As described previously, an optimal CPR could improve survival after CA (3, 6, 7), however it could be harder to determine laypeople's CPR quality during CPR training without QCPR device. In this study, performance score obtained automatically and with the checklist were correlated, and MCPR scores appeared predictive of CPR quality defined by QCPR global-Scores over 50 or 75%. This result was consistent with the literature that objective and detailed checklist were sufficient to assess CPR quality (38).

This study showed that CPR performance was correlated to laypeople's CPR theoretical knowledge after didactic reminder but not after practice training. However, after the didactic reminder, performance scores remained low. This result highlighted that improved theoretical knowledge in CPR was not enough to obtain a satisfactory CPR quality and the interest of practical training (39, 40). Moreover, as presented in this study, the association of didactic intervention and SBT—mastery training—could maintain knowledge and skills up to 2 months as for healthcare providers (13, 14, 29, 32, 41) which correspond to an adequate which corresponds to an adequate amount of time to repeat the training or assessment of the performance (42, 43).

### Limitations

This study had some limitations. First, while the inclusion rate was high number of participants included was small leading to little group to assess retention over time. However, the results obtained by the three groups were very similar and it seems unlikely that the lack of difference is related to a lack of statistical power.

Secondary, the equipment used for simulation was only presented during briefing before CPR performance while most of participant had never used QCPR devices or manikin Resusci Baby QCPR before the study. The lack of awareness of the possibilities of these elements by the participants may had influenced results of E1 and artificially increased those of E2. However, the influence of equipment on score may be low because all participants were previously trained to CPR on different types of manikin. The use of the same scenario for E1 and E2 may have contributed to artificially increasing the results on E2. However, before E2 and until the briefing, participants were not informed that scenario used for E2 would be the same as for E1 to prevent external training. Moreover, using the same scenario was necessary to compare performance.

Another limitation of the study was the absence of evaluation before any intervention to know CPR performance at baseline and to correlate it to theoretical knowledge. However, score and skills at E1 were low and were probably not or only slightly modified by the didactic intervention. Moreover, previous studies have demonstrated the low quality of CPR performance of laypeople at baseline (8, 9). The CPR sequence used was A-B-C and not C-A-B because it dealt with infants. Comparison of performance between groups using a different sequence may be interesting but the small number of participants limited this possibility but could be the purpose of a future study.

The used of non-standardized checklist was a limitation of the study however the checklist was developed from ERC recommendations and allowed to assess skills unrecorded by the QCPR device. Moreover, MCPR score determined with the checklist was strongly correlated to the CPR quality assess automatically with the QCPR device. The knowledge questionnaire was also non-standardized however no standardized questionnaire was previously available. In addition, each answer can be deduced from the didactic reminder performed at T1 and ensured that the participant had sufficient knowledge to perform a CPR. In addition,

### External Validity

To our knowledge, this is the first simulation-based study about infant CPR performance of nursery assistants working alone at home proposing an easy and accessible method to assess infant CPR quality. The chosen population included motivated and very engaged nursery assistant women previously trained once every 2 years in infant CPR. In addition to the small number of participants, it would be difficult to generalize these results to all of the nursery assistant, other caregivers or laypeople and further study with a larger number of participants would be necessary.

## Conclusion

Mastery training including didactics and SBT could improve performance, skills, knowledge with satisfactory retention over time in infant CPR management performed by nursery assistants especially for chest compression step. CPR performance quality could be assessed with detailed CPR checklist. This training represents an interesting method to train laypeople in charge of infants in management of different emergencies requiring immediate support while waiting for help. However, further study would be useful in determining the number of SBT training sessions required for each participant to achieve advanced quality. The repetition of simulation sessions would be mandatory for long-term memory retention as infant cardiac arrest represents a low volume-high stakes situation. Further studies should include larger area-based populations and measure critical events management in infants taken care of by nursery assistants.

## Data Availability Statement

The datasets generated for this study are available on request to the corresponding author.

## Ethics Statement

Ethical review and approval was not required for the study on human participants in accordance with the local legislation and institutional requirements. The patients/participants provided their written informed consent to participate in this study.

## Author Contributions

Conception and design of the study, or acquisition of data, or analysis and interpretation of data: FB, BB, VG-M, and DO. Drafting the article or revising it critically for important intellectual content: FB, AG, and DO. All authors contributed to the article and approved the submitted version.

### Conflict of Interest

The authors declare that the research was conducted in the absence of any commercial or financial relationships that could be construed as a potential conflict of interest.

## Abbreviations

AHA: American Heart Association
ALTE: Apparent life-threatening event
BLS: Basic life support
CA: Cardiac arrest
CPR: Cardiopulmonary resuscitation
OHCA: Out-of-hospital cardiac arrest
ROC: Receiver operating characteristic
SBT: Simulation-based training
SD: standard deviation.
