# Supplementary figures and images for: Nursery Assistants' Performance and Knowledge on Cardiopulmonary Resuscitation: Impact of Simulation-Based Training
Source: Front Pediatr. 2020 Jun 30;8:356. doi: 10.3389/fped.2020.00356 (PMC7338941; doi:10.3389/fped.2020.00356)

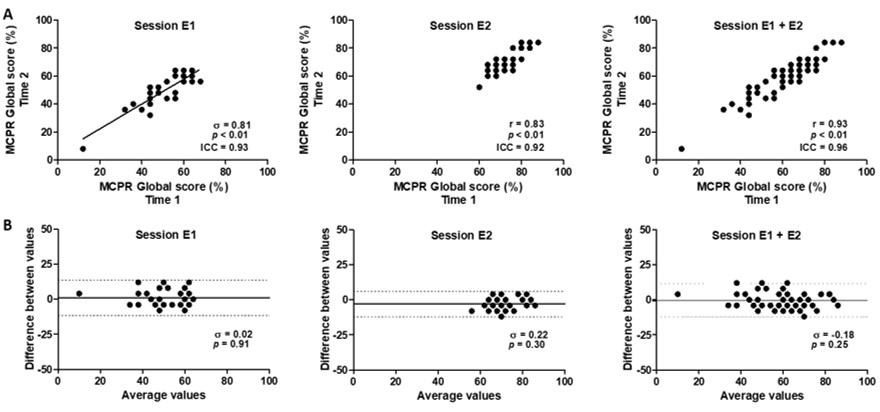

Supplement: Supplementary file 1 [file Image_1.JPEG]

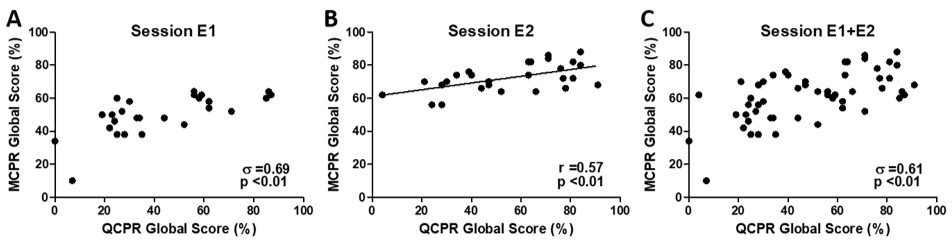

Supplement: Supplementary file 2 [file Image_2.JPEG]

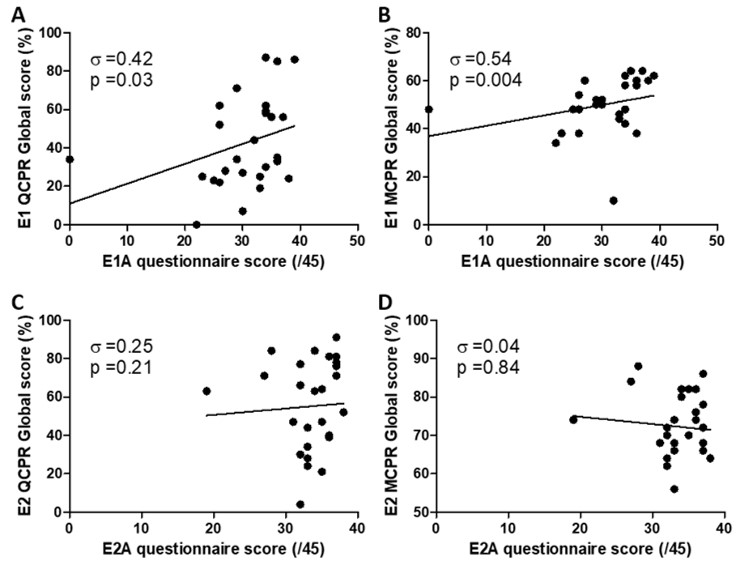

Supplement: Supplementary file 3 [file Image_3.JPEG]
