# Supplementary material for: Nursery Assistants' Performance and Knowledge on Cardiopulmonary Resuscitation: Impact of Simulation-Based Training
Source: Front Pediatr. 2020 Jun 30;8:356. doi: 10.3389/fped.2020.00356 (PMC7338941; doi:10.3389/fped.2020.00356)
Supplement: Supplementary file 4 [file Data_Sheet_1.docx]

**Online supplemental data**

**Nursery assistants’ performance and knowledge on cardiopulmonary resuscitation: impact of simulation-based training.**

Fabien Beaufils^1,2^, Aiham Ghazali^1,3^, Bettyna Boudier^1^, Valérie Gustin-Moinier^4^, Denis Oriot^1,5^

**Supplemental figure legends**

**Figure E1: Correlation of MCPR score assessement**

A) Graphs of data from correlation over time for MCPR-Score at E1, E2 and for all values (left, middle, and right graphs, respectively). B) Graphs of means of measurements over time for MCPR-score at E1, E2 and for all values (left, middle, and right graphs, respectively) are plotted against their difference according to Bland-Altman analysis. Solid lines correspond to the mean difference. Dashed lines correspond to the mean difference + 2 standard deviations and the 95% confidence interval. ICC = intraclass correlation coefficient. *p* value <0.05 was considered significant. r: Pearson’s correlation coefficient; σ: Spearman’s correlation coefficient.

**Figure E2: Correlations between QCPR Global-scores and MCPR Global-Scores**

Correlations between QCPR Global-scores and MCPR Global-Scores obtained at A) E1, B) E2 and C) for all values together. Pearson test was used to assess correlation for parametric values or Spearman test for non-parametric values. r: Pearson’s correlation coefficient; σ: Spearman’s correlation coefficient.

**Figure E3: Correlation between theoretical knowledge score and QCPR or MCPR Global-Scores**

Correlation between theoretical knowledge score obtained at the beginning of session E1 and A) the QCPR score or B) the MCPR score obtained at E1. Correlation between theoretical knowledge score obtained at the beginning of session E2 and C) the QCPR score or D) the MCPR score obtained at E2. Pearson test was used to assess correlation for parametric values or Spearman test for non-parametric values. r: Pearson’s correlation coefficient; σ: Spearman’s correlation coefficient.

**Supplemental TableS**

| **Table E1 Receiver operating characteristic curve analyses of the MCPR Global-Score ability to predict QCPR Global Score ≥50 or QCPR Global Score ≥75%** | | | | | | | | |
| --- | --- | --- | --- | --- | --- | --- | --- | --- |
|  |  | **AUC** | **AUC’s SE** | ***p* value** | **Sn** | **Sp** | **PPV** | **NPV** |
| QCPR Global Score ≥50% | | 0.77 | 0.06 | <0.01 | 0.96  (0.80;1.00) | 0.39  (0.22;0.60) | 0.60  (0.43;0.74) | 0.92  (0.62;0.99) |
| QCPR Global Score ≥75% | | 0.77 | 0.07 | <0.01 | 0.36  (0.11;0.69) | 0.88  (0.75;0.96) | 0.44  (0.14;0.79) | 0.84  (0.71;0.94) |
| Data are absolute number with 95% CI  AUC: area under curve; Sn: Sensitivity; Sp: Specificity; PPV: Positive predictive value; NPV: Negative predictive value; QCPR Global Score: CPR performance score assessed with SimPad PLUS SkillReporter; MCPR Global Score: CPR performance score assessed with checklist.. Sn, Sp, PPV, NPV of MCPR Global Score to predict QCPR Global Score ≥50 or ≥75 was determined using a cut off value of each score at 50% or 75% (corresponding to MCPR global score >12/25 or ≥18) | | | | | | | | |

| Table E2 – Persistence of performance over time | | | | |
| --- | --- | --- | --- | --- |
|  | **Group 1 D15**  **N=9** | **Group 2 D30**  **N=9** | **Group 3 D60**  **N=9** | ***p* value** |
| ***QCPR Global-Score (%)*** | 59.0 [24.5;74.0] | 71.0 [39.0;76.5] | 63.0 [34.5;79.5] | 0.887 |
| ***MCPR Global-Score (%)*** | 76.0 [70.0;83.0] | 72.0 [67.0;80.0] | 68.0 [63.0;73.0] | 0.125 |
| ***AIRWAYS step*** |  |  |  |  |
| Stimulate infant | 8/1 | 9/0 | 8/1 | 0.582 |
| Check breath | 7/2 | 5/4 | 6/3 | 0.223 |
| Put on hard surface | 9/0 | 9/0 | 9/0 | 1.000 |
| Put in neutral position | 7/2 | 7/2 | 6/3 | 0.825 |
| Opening airway | 5/4 | 7/2 | 5/4 | 0.530 |
| Inspecting airways | 5/4 | 4/5 | 4/5 | 0.862 |
| ***COMMUNICATION step*** |  |  |  |  |
| Calling emergency medical system | 9/0 | 9/0 | 9/0 | 1.000 |
| Continue chest compression during call | 8/1 | 7/2 | 7/2 | 0.587 |
| ***BREATHING step*** |  |  |  |  |
| QCPR Breathing Score (%) | 55.0 [51.5;79] | 59.0 [53.0;73.0] | 50.0 [50.0;80.0] | 0.783 |
| Time of first rescue breath (sec) | 47.0 [38.5;91.8] | 45.0 [38.0;51.5] | 48.5 [33.0;59.0] | 0.796 |
| Ventilation with correct volume (%) | 9.0 [0.0;16.8] | 11.0 [0.0;28] | 8.0 [0.0;31.0] | 0.854 |
| Volume over the maximum volume (%) | 91.0 [48.3;100] | 80.0 [55.5;97.0] | 96.0 [31.8;100] | 0.931 |
| Volume below the minimum volume (%) | 6.0 [0.0;15.5] | 10.5 [0.0;26.5] | 0.0 [0.0;31.0] | 0.821 |
| Starting ventilation before chest compression (yes/no) | 9/0 | 9/0 | 9/0 | 1.000 |
| 5 Rescue breaths before chest compression (yes/no) | 8/1 | 8/1 | 9/0 | 0.587 |
| Rescue breaths with long duration (yes/no) | 1/8 | 1/8 | 1/8 | 1.000 |
| Chest expansion observed during rescue breathes (yes/no) | 9/0 | 7/2 | 8/1 | 0.325 |
| ***COMPRESSION/CIRCULATION step*** |  |  |  |  |
| QCPR Compression Score (%) | 70.0 [9.0;80] | 80.0 [23.0;84.0] | 74.0 [10.5;95] | 0.800 |
| Time of first compression (sec) | 66.0 [55.5;89.0] | 64.0 [52.5;75.0] | 63.0 [50.5;73.0] | 0.783 |
| Compressions with correct rate (%) | 46.0 [7.5;77.5] | 49.0 [27.5;68.5] | 57.0 [38.5;79.5] | 0.643 |
| Participant with rate between100-120/min (Yes/No) |  |  |  |  |
| Compressions with correct hand position (%) | 86.0 [31.5;97.5] | 78.0 [47.0;99.5] | 84.0 [57.5;96.5] | 0.957 |
| Compressions with correct depth (%) | 73.0 [24.5;90.5] | 86.0 [70.5;95.0] | 92.0 [23.0;98.0] | 0.572 |
| Chest compressions correctly recalled (%) | 82.0 [65.0;99.5] | 87.0 [40.0;99.0] | 95.0 [69.0;99.0] | 0.963 |
| No flow duration during the session (sec) | 81.0 [69.5;105.5] | 78.0 [69.5;95.5] | 81.0 [59.0;89.5] | 0.813 |
| No flow duration during the session (%) | 36.4 [30.0;41.7] | 35.3 [29.8;43.3] | 40.1 [27.2;43.8] | 0.98 |
| No flow duration after the first compression (sec) | 13.0 [11.5;16.5] | 15.0 [12.5;22.0] | 15.0 [10.5;19.0] | 0.693 |
| Starting chest compressions (Yes/No) | 9/0 | 9/0 | 9/0 | 1.000 |
| Maintaining airway in open position (Yes/No) | 2/7 | 3/6 | 1/8 | 0.526 |
| Using two fingers technique for compressions (Yes/No) | 7/2 | 7/2 | 6/3 | 0.825 |
| With good position of finger on chest (Yes/No) | 6/3 | 5/4 | 7/2 | 0.607 |
| Correct chest dept (Yes/No) | 7/2 | 8/1 | 8/1 | 0.746 |
| Performing compressions-breath 30:2 (Yes/No) | 9/0 | 8/1 | 9/0 | 0.354 |
| Questionnaire E2A Global Score /45 | 34.0 [29.5;35.5] | 33.0 [31.5;36.5] | 35.0 [32.5;37.0] | 0.546 |
| QCPR Breathing-Score and QCPR Compression-Score represented scores assessed automatically with SimPad PLUS SkillReporter.  Data are median [IQR_25_;IQR_75_] deviation for quantitative values. Comparisons of continuous variables were made with Paired T-test for parametric variables or Wilcoxon test for non-parametric variables. Categorial variables were analyzed with Fisher’s exact test. | | | | |
