# Supplementary material for: Nursery Assistants' Performance and Knowledge on Cardiopulmonary Resuscitation: Impact of Simulation-Based Training
Source: Front Pediatr. 2020 Jun 30;8:356. doi: 10.3389/fped.2020.00356 (PMC7338941; doi:10.3389/fped.2020.00356)
Supplement: Supplementary file 5 [file Data_Sheet_2.docx]

| **Scenario**  “You are at home on a Tuesday afternoon. You are taking care of a 2-month old infant called Simon.  The parents dropped him off this morning and told you that he had just picked up a small cold. He coughed slightly during the morning and ate less than usual.  At naptime, you put him in his bed where he fell asleep quickly. Concerned about his health condition, you left a baby phone next to him. After 30 minutes of nap, you heard him through the baby phone coughing intensely with noisy breathing and suddenly no sound came out of the baby phone.  You quickly went to his room and discovered a pale child with blue lips.  We ask you, once you are behind the door, to act as you would in a real situation. The simulation will end at my signal.”  Algorithm expected =Cardiopulmonary arrest at home in a 2-month-old infant  - Stimulation, positioning in neutral position, opening and securing airway, if unresponsive  - Start ventilation, if no sign of life after ventilation  - Start compressions, if no sign of life after 5 cycles 2/30 (1 min of RCP)  - Call emergency (# 15, 112 or 18) and maintain CPR during the call |
| --- |

**Appendix A1**: Scenario

| **CPR PERFORMANCE CHECKLIST** | | |
| --- | --- | --- |
| **Name or ID: Date: / /** | | Participant’s score |
| AIRWAYS | |  |
| Stimulate infant (Yes = 1 point, No = 0 point) | |  |
| Check breath (Yes = 1 point, No = 0 point) | |  |
| Put on hard surface (Yes = 1 point, No = 0 point) | |  |
| Put in neutral position (Yes = 1 point, No = 0 point) | |  |
| Open and secure airway (Both = 2 points, once=1 point, none =0 point) | |  |
|  | Total of AIRWAYS steps  (MCPR Airway-Score) | /6 |
| BREATHING | |  |
| Start rescue breaths before chest compression (Yes = 1 point, No = 0 point) | |  |
| Number (5 rescue breathes = 2 points; <5 = 1 point, 0 = 0 point) | |  |
| Duration (Long = 2 points, short = 1 point, no rescue breath = 0 point) | |  |
| Quality (Chest ampliation = 1 point; no ampliation or no rescue breath = 0 point) | |  |
|  | Total of BREATHING steps  (MCPR Breathing-Score) | /6 |
| CIRCULATION | |  |
| Start chest compressions (Yes = 1 point, No = 0 point) | |  |
| Maintain head in neutral position during compression (Yes = 1 point, No = 0 point) | |  |
| Use 2 fingers technique for compression (Yes = 1 point, No = 0 point) | |  |
| Finger position on chest correct (Yes = 1 point, No = 0 point) | |  |
| Chest dept ≈ 4 cm (Yes = 1 point, No = 0 point) | |  |
| Rate (100-120/min = 2 points, 80-99 or 121-140 = 1 point, <80 or > 140 = 0 point) | |  |
| Alternate with ventilation (after 30 chest compressions = 2 points, >30 = 1 point; < 30 = 0 points) | |  |
|  | Total of CIRCULATION steps  (MCPR Compression-Score) | /9 |
| COMMUNICATION | |  |
| Number of cycles before emergency call (5 cycles = 2 points, <5 = 1 point, no call = 0 point) | |  |
| Continue compression during call (correctly =2 points, incorrectly = 1 point; not continue = 0 point) | |  |
|  | Total of COMMUNICATION steps  (MCPR Communication-Score) | /4 |
|  | Total of BREATHING and CIRCULATION steps  (MCPR BC-Score) | /15 |
|  | TOTAL of all part  (MCPR Global-Score) | /25 |

**Appendix A2:** CPR performance checklist used to assess CPR performance manually

| **Questionnaire**   \| What are the signs of apparent life-threatening event in infant? /7 \| \| \| \| \| \| \| \| \| \| --- \| --- \| --- \| --- \| --- \| --- \| --- \| --- \| --- \| \| No spontaneous movement ………………………………………………….. \| \| \| \| \| \| \| □ Yes \| □ No \| \| No spontaneous breath ……………………………………………………… \| \| \| \| \| \| \| □ Yes \| □ No \| \| Apnea over 10 seconds ……………………………………………………… \| \| \| \| \| \| \| □ Yes \| □ No \| \| Pale skin color ………………………………………………………………. \| \| \| \| \| \| \| □ Yes \| □ No \| \| Tone disorder ………….…………………………………………………… \| \| \| \| \| \| \| □ Yes \| □ No \| \| Heart rate below 60/min …………………………………………………….. \| \| \| \| \| \| \| □ Yes \| □ No \| \| Presence of mottling ……………………………………………………… \| \| \| \| \| \| \| □ Yes \| □ No \| \| About infant cardiac arrest /6 \| \| \| \| \| \| \|  \|  \| \| Is frequently caused by a heart disease, heart rhythm disorder ……………… \| \| \| \| \| \| \| □ Yes \| □ No \| \| Is frequently caused by a respiratory disease ……………………………….. \| \| \| \| \| \| \| □ Yes \| □ No \| \| Affects more frequently children over six months ………………………….. \| \| \| \| \| \| \| □ Yes \| □ No \| \| Always needs an urgent care at home ……………………………………….. \| \| \| \| \| \| \| □ Yes \| □ No \| \| Increases mortality risk if happened at home………………………………… \| \| \| \| \| \| \| □ Yes \| □ No \| \| Mortality or sequalae are increased without management in the 10 first min \| \| \| \| \| \| \| □ Yes \| □ No \| \| Which action is required for apparent life -threatening event in infant with cardiac arrest? /6 \| \| \| \| \| \| \| \| \| \| Completely undress infant…………………………………………………….. \| \| \| \| \| \| \| □ Yes \| □ No \| \| Check infant reactivity………………………….…………………………….. \| \| \| \| \| \| \| □ Yes \| □ No \| \| Check pulse…………………………………………………………………… \| \| \| \| \| \| \| □ Yes \| □ No \| \| One rescuer, put infant on hard surface……………………………….………. \| \| \| \| \| \| \| □ Yes \| □ No \| \| Two rescuers, put infant on hard surface………………….…………………... \| \| \| \| \| \| \| □ Yes \| □ No \| \| Wait for help by watching the child without action…………………………... \| \| \| \| \| \| \| □ Yes \| □ No \| \| Order actions in management of CPR with alone rescuer: /6 \| \| \| \| \| \| \|  \|  \| \| Inspection of mouth and secure airways………………………………………. \| \| \| \| \| \| \| _ \| \| \| Start chest compressions………….………………………………….………... \| \| \| \| \| \| \| _ \| \| \| Start ventilation……. …………………………………………………………. \| \| \| \| \| \| \| _ \| \| \| Stimulate the infant……………………………………………………………. \| \| \| \| \| \| \| _ \| \| \| Call emergency………………………………………………………………... \| \| \| \| \| \| \| _ \| \| \| Secure the scene………………………………………………………………. \| \| \| \| \| \| \| _ \| \| \| About chest compression in infant CPR: /5 \| \| \| \| \| \| \|  \|  \| \| Compression rate is the same as in adults…………………….………………. \| \| \| \| \| \| \| □ Yes \| □ No \| \| Correct compression rate is between 100 and 120 compressions/min………... \| \| \| \| \| \| \| □ Yes \| □ No \| \| With two rescuers, compression/ventilation ratio is……… \| \| \| \| \| □ 20:1 \| □ 15:2 \| □ 30:2 \| □ 15:1 \| \| With one rescuer, compression/ventilation ratio is………. \| \| \| \| \| □ 20:1 \| □ 15:2 \| □ 30:2 \| □ 15:1 \| \| Chest compression depth in CPR is …………… \| \| \| \| □ 1cm \| □ 2cm \| □ 3cm \| □ 4cm \| □ 5cm \| \| About ventilation in infant CPR: /4 \| \| \| \|  \|  \|  \|  \|  \| \| The number of rescue breaths is……... \| □ 0 \| \| \| □ 1 \| □ 2 \| □ 3 \| □ 4 \| □ 5 \| \| Is not recommended in infant CPR……………….…………………………… \| \| \| \| \| \| \| □ Yes \| □ No \| \| Is recommended if there is only one rescuer………………………………….. \| \| \| \| \| \| \| □ Yes \| □ No \| \| Is recommended if there are two rescuers…………….………………………. \| \| \| \| \| \| \| □ Yes \| □ No \| \| EMS rescuers must be called: /11 \| \| \| \| \| \| \|  \|  \| \| At the phone number (3 answers attended)……. \| \| \| □ 112 \| \| □ 14 \| □ 15 \| □ 17 \| □ 18 \| \| Before starting CPR if there is only one rescuer (/2) ………………………… \| \| \| \| \| \| \| □ Yes \| □ No \| \| One minute after starting CPR………………………………………………… \| \| \| \| \| \| \| □ Yes \| □ No \| \| After X cycles of CP/V……………….. \| \| □ 0 \| \| □ 1 \| □ 2 \| □ 3 \| □ 4 \| □ 5 \| \| Before CPR with two rescuers (/2).…………………………………………... \| \| \| \| \| \| \| □ Yes \| □ No \| \| Five minutes after starting CPR with two rescuers.…………………………... \| \| \| \| \| \| \| □ Yes \| □ No \| \| A communication technique exists to sum up situation to EMS rescuers..…… \| \| \| \| \| \| \| □ Yes \| □ No \| |
| --- | --- | --- | --- | --- | --- | --- | --- | --- | --- | --- | --- | --- | --- | --- | --- | --- | --- | --- | --- | --- | --- | --- | --- | --- | --- | --- | --- | --- | --- | --- | --- | --- | --- | --- | --- | --- | --- | --- | --- | --- | --- | --- | --- | --- | --- | --- | --- | --- | --- | --- | --- | --- | --- | --- | --- | --- | --- | --- | --- | --- | --- | --- | --- | --- | --- | --- | --- | --- | --- | --- | --- | --- | --- | --- | --- | --- | --- | --- | --- | --- | --- | --- | --- | --- | --- | --- | --- | --- | --- | --- | --- | --- | --- | --- | --- | --- | --- | --- | --- | --- | --- | --- | --- | --- | --- | --- | --- | --- | --- | --- | --- | --- | --- | --- | --- | --- | --- | --- | --- | --- | --- | --- | --- | --- | --- | --- | --- | --- | --- | --- | --- | --- | --- | --- | --- | --- | --- | --- | --- | --- | --- | --- | --- | --- | --- | --- | --- | --- | --- | --- | --- | --- | --- | --- | --- | --- | --- | --- | --- | --- | --- | --- | --- | --- | --- | --- | --- | --- | --- | --- | --- | --- | --- | --- | --- | --- | --- | --- | --- | --- | --- | --- | --- | --- | --- | --- | --- | --- | --- | --- | --- | --- | --- | --- | --- | --- | --- | --- | --- | --- | --- | --- | --- | --- | --- | --- | --- | --- | --- | --- | --- | --- | --- | --- | --- | --- | --- | --- | --- | --- | --- | --- | --- | --- | --- | --- | --- | --- | --- | --- | --- | --- | --- | --- | --- | --- | --- | --- | --- | --- | --- | --- | --- | --- | --- | --- | --- | --- | --- | --- | --- | --- | --- | --- | --- | --- | --- | --- | --- | --- | --- | --- | --- | --- | --- | --- | --- | --- | --- | --- | --- | --- | --- | --- | --- | --- | --- | --- | --- | --- | --- | --- | --- | --- | --- | --- | --- | --- | --- | --- | --- | --- | --- | --- | --- | --- | --- | --- | --- | --- | --- | --- | --- | --- | --- | --- | --- | --- | --- | --- | --- | --- | --- | --- | --- | --- | --- | --- | --- | --- | --- | --- | --- | --- | --- | --- | --- | --- | --- | --- | --- | --- | --- | --- | --- | --- | --- | --- | --- | --- | --- | --- | --- | --- | --- | --- | --- | --- | --- | --- | --- | --- | --- | --- | --- | --- | --- | --- | --- | --- | --- | --- | --- | --- | --- | --- | --- | --- | --- | --- | --- | --- | --- | --- | --- | --- | --- | --- | --- | --- | --- | --- | --- | --- | --- | --- | --- | --- | --- | --- | --- | --- | --- | --- | --- | --- | --- | --- | --- | --- | --- | --- | --- | --- | --- | --- | --- | --- | --- | --- | --- | --- | --- | --- | --- | --- | --- | --- | --- | --- | --- | --- | --- | --- | --- | --- | --- | --- | --- | --- | --- | --- |

**Appendix A3:** Theoretical knowledge questionnaire used at the beginning (A) and the end (B) of session T1 (QT1A/B), Session E1 (QE1A/B), Session E2 (QE2A/B). Each good answer represents 1 point for a maximum of 45 points.
